# Valorisation of Beetroot Peel for the Development of Nutrient-Enriched Dehydrated Apple Snacks

**DOI:** 10.3390/foods14152560

**Published:** 2025-07-22

**Authors:** Ioana Buțerchi, Liliana Ciurlă, Iuliana-Maria Enache, Antoanela Patraș, Gabriel-Ciprian Teliban, Liviu-Mihai Irimia

**Affiliations:** “Ion Ionescu de la Brad” Iasi University of Life Sciences, 3 Mihail Sadoveanu, Alley, 700490 Iasi, Romania; ioana.buterchi@iuls.ro (I.B.); liliana.ciurla@iuls.ro (L.C.); iuliana.enache@iuls.ro (I.-M.E.); antoanela.patras@iuls.ro (A.P.); gabriel.teliban@iuls.ro (G.-C.T.)

**Keywords:** vegetable by-products, beetroot peel, nutritional value, bioactive compounds, apple snacks, functional food

## Abstract

Beetroot peel, an underutilised by-product of the food industry, has significant potential for valorisation due to its high content of bioactive compounds and natural pigments. This study aimed to sustainably reintroduce beetroot peel into the food chain by enriching the nutritional value of dehydrated apple snacks. Five experimental formulations of apple slices were developed: dipped in 5% RBPP in water, dipped in 10% RBPP in water, dipped in 5% RBPP in 50% lemon juice, dipped in 10% RBPP in 50% lemon juice all seasoned with cinnamon powder, and a control formulation. The biochemical analysis showed that the total phenolic content (2780.01 ± 68.38 mg GAE/100 g DM) and antioxidant activity of apple snacks significantly increased (503.96 ± 1.83 µmol TE/g DM). Sensory evaluation indicated that snacks with beetroot peel powder and lemon juice achieved the highest scores in colour, flavour, and acceptability. These results demonstrate that the valorisation of beetroot peel has the potential to reduce agro-industrial waste and also enhance the nutritional and functional quality of apple snacks. It is recommended that beetroot peel be further explored as a cost-effective natural ingredient to develop healthier, value-added snack products within a circular economy framework.

## 1. Introduction

The valorisation of food waste is increasingly recognised as a critical component of sustainable development within a circular economy model [1]. The recovery of fruit and vegetable waste and by-products such as peels, seeds, or pomace can fulfil modern food industry requirements for producing natural pigments (anthocyanins, betalains, carotenoids, chlorophylls, etc.) and new sources of nutrients. Recognising the imperative to safeguard the environment, secure future food supplies, and preserve resources, sustainability has emerged as a crucial focus within the food industry [2,3]. A sustainable way of waste management is the recovery of the bioactive compounds from these by-products and reuse them in new value-added food products. Thus, the agro-food waste is reduced and the environment and the health of biocenosis are protected [4,5,6].

Red beetroot peel is an example of an agro-food by-product that can be reintroduced in the food supply chain to obtain enriched products, mainly due to its high content of bioactive compounds and pigments [7]. This high amount of valuable compounds from red beetroot peel is reflected in its antioxidant activity, which was found to be higher than that of other vegetable [8]. Compared to many frequently eaten vegetables’ by-products, such as carrot and celery (without antioxidant activity), kale (108 mg Trolox kg^−1^ fw), and broccoli (420 mg Trolox kg^−1^ fw) [9], beetroot antioxidant activity is either higher or equivalent. Beetroot peel is a strong, undervalued source of antioxidants that could substantially enhance the functional qualities of food products like apple snacks, which strengthens the case for its valorisation. Beetroot peel is a strong, undervalued source of antioxidants that could substantially enhance the functional qualities of food products like apple snacks, which strengthens the case for its valorisation. Furthermore, the peel of beetroot has demonstrated notable antibacterial activity [10,11]. During industrial processing, beetroots are peeled, and the waste skins, about 11–50% of the root, are eliminated [12]. Red beetroot (*Beta vulgaris* L.) is an important crop, valued for its rich phytochemical profile, which can be valorised in the food or pharmaceutical industry, cosmetics, etc. The characteristic crimson hue of beetroot is attributed to high concentrations of betalains [13] natural pigments increasingly explored in the food industry [14] for their potential human health benefits, particularly anti-inflammatory effects [15]. Predominantly, betacyanins and betaxanthins constitute the betalain content of beetroots [16,17].

Global product volumes demonstrate that apples are among the most popular fruits, and their value in the human nutrition is widely established [18,19]. Apples naturally include a variety of minerals, organic acids, and vitamins, as well as carbohydrates and dietary fibre (of which soluble fibre makes up 80%) [19]. The nutritional significance of fresh apples (*Malus domestica*) comes from their balanced chemical profile and the high bioavailability of their constituents, coupled with desirable organoleptic properties [20,21,22]. Their prolonged shelf life under optimal storage conditions, along with their adaptability to various processing techniques, including drying or other processes, enhances their nutritional significance [23].

In order to introduce innovative, safer, fresher, and higher-quality foods with a longer shelf-life for both domestic and international markets, researchers are combining food processing technology and recipes [24]. According to Sobukola et al. (2006) [25] and Çoklar et al. (2017) [26], one of the earliest methods of food preservation that humans have ever used is the drying of fruits and vegetables. This method prolongs the shelf life of dried products by decreasing deteriorative chemical processes and offers microbiological stability by reducing biologically active water to a safe level [27]. Dehydration is a food technology that can be used to extend the shelf life and quality maintenance of apples [28], thus reducing food waste while fulfilling the requirements of modern consumers for healthy, less processed, but also higher-quality foods [29]. Also, dehydration can serve as an alternative to freezing or chemical preservation, or it can be used alongside these techniques as a straightforward, safe, and cost-effective approach that helps maintain nutrient content [30].

As natural and rich sources of sugars, vitamins A and C, niacin, riboflavin, folic acid, potassium, other oligoelements, iron, copper, and organic acids as well as phytonutrients with antioxidant qualities, dried apples are widely recognised for their nutritional and dietary qualities. A low glycaemic index [31,32] and dietary fibre content, especially insoluble fibre, are two of their many significant qualities.

Current research valorises underutilised apple and beetroot processing residues to formulate a clean-label, multifunctional dried apple snack that is remarkably rich in antioxidants (phenolics, flavonoids, anthocyanins, betalains, and vitamin C) and stabilised in colour (lemon-induced enzymatic inhibition and beetroot pigments). Moreover, through dehydration, the extended shelf life of products and enhanced sensory appeal can be achieved without synthetic additives, aligning with emerging valorisation strategies that aim to convert fruit and vegetable waste into high-value functional foods.

This food product is addressed to the modern consumer whose interest in healthy food products is growing, as they are aware of the impact of what they consume on their health. Also, Testa et al. (2023) [33] show that the consumer is increasingly aware of the need for a sustainable and environmentally friendly food product, taking into account the limited resources of the planet. Sabbe et al. (2008) [34] demonstrated that product familiarity is the primary factor influencing purchase intention, whereas Jesionkowska et al. (2009) [35] emphasised that health considerations are the primary factors influencing the selection of dried fruits that are thought to be high in functional ingredients. Thus, challenges for the food industry include providing healthy and sustainable food products [6]. The use of beetroot by-products in food applications, including beverages, baked products, functional snack bars, and expanded grain snacks, has been studied in the past, but no study has explicitly looked at fortifying fruit-based snacks with beetroot peel powder, especially in dehydrated apple formats. The complementary effects of beetroot peel and lemon juice on the physicochemical, sensory, and nutritional aspects of apple snacks are also unknown. In this sense, it was decided to obtain and analyse this type of product. Furthermore, the enriched apple snack obtained in this study supports consumers to choose simple and natural alternatives, without artificial dyes or preservatives, obtained using natural ingredients and through a sustainable method.

## 2. Materials and Methods

### 2.1. Materials and Reagents

The base for the new food product is represented by fresh and healthy apples from Idared cultivar, red beetroot, lemon and cinnamon. Idared apples (diameter 70–75 mm, average weight 140 ± 10 g) were purchased from the experimental orchard of the IULS, in Iași county (47.194325 N long. and 27.547779 E long.) and selected for their uniform ripeness and absence of defects. Fresh medium-sized red beets were purchased from the local market in Iași, with a diameter of 45–55 mm and a weight of ~110 g, washed and cleaned manually to separate the peel (thickness ~3 mm). Fresh medium-sized lemons, with a diameter of 50–60 mm, purchased from the market in Iași, yielded ~35 mL of juice each after being cut in half and squeezed manually.

The extraction procedure was performed using ultrapure water, HPLC-grade methanol, and hydrochloric acid 37%. For the physicochemical analysis, the following analytical-grade reagents were used: 0.1 N NaOH solution, Folin–Ciocalteu reagent, sodium carbonate, gallic acid, 2,2-diphenyl-1-picrylhydrazyl (DPPH), and 6-hydroxy-2.5.7.8-tetramethylchroman-2-carboxylic acid (Trolox). Acetonitrile, trifluoroacetic acid, gallic acid, protocatechuic acid, *p*-hydroxybenzoic acid, vanillic acid, caffeic acid, catechin, chlorogenic acid, vanillin, syringic acid, coumaric acid, epicatechin, ferulic acid, salicylic acid, sinapic acid, rosmarinic acid, resveratrol, and quercetin were the HPLC-grade reagents used for the HPLC analysis. All the used reagents were purchased from Sigma-Aldrich (Steinheim, Germany).

### 2.2. Processing Raw Ingredients

**Figure 1:**
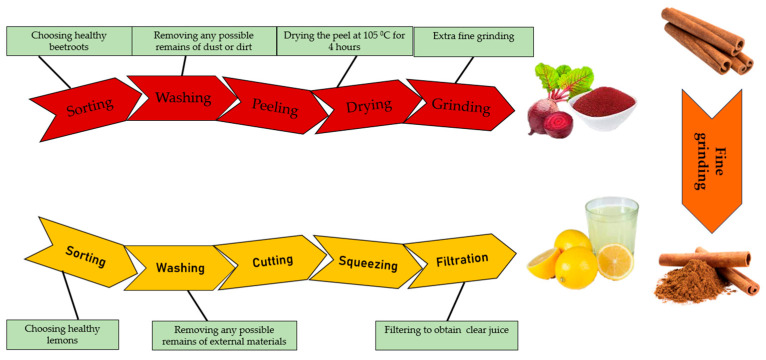
Experimental protocol for obtaining red beetroot peel powder, lemon juice and cinnamon powder.

The red beetroot peel powder (RBPP) used in the preparation of the dried apple snack was obtained via the following steps (Figure 1):

✓ Selection and Sorting: We chose beetroots that were fresh and healthy, avoiding any that were overripe, mouldy, or damaged. Only healthy, well-grown roots were processed.
✓ Cleaning: To remove any dust or debris, beetroots were thoroughly cleaned under the pressure of the water.
✓ Peeling: The root peel was removed using a hygienic scraper, eliminating damaged or discoloured portions to ensure consistent quality.
✓ Peel preparation: The clean peel was spread in a single layer on stainless steel trays, with each piece of peel being approximately 3 mm thick, in accordance with the recommendations in the specialist literature for optimal drying.
✓ Drying: Trays were placed in a controlled hot-air chamber at 38 °C for six hours, until the peels were fully dehydrated and crisp.
✓ Grinding: The beetroot peel was first dried thoroughly and then ground in a high-speed grinder until a fine, homogeneous powder was obtained. A subsequent stage of sieving through a fine sieve ensured the removal of oversized particles, resulting in particle sizes with values less than 40 µm, suitable for our experiment.
✓ Packaging: The resulting fine powder was immediately transferred to hermetic moisture-resistant bags (zipper system) to prevent moisture absorption.
✓ Storage: Packaged powder was stored in a cool, dark area (to prevent degradation from light exposure) with low relative humidity to maintain stability of colour and bioactive compounds.

The lemon juice was obtained via the following procedure: sorting the lemons, choosing only healthy and fresh lemons; washing the lemons to remove any dirt or dust; cutting; squeezing; and finally, filtration to obtain clear juice. The cinnamon was bought in the form of sticks and ground to a fine powder to season the snacks. The experimental protocol was carried out by following the steps presented in Figure 1.

### 2.3. Red Beetroot Peel Powder Characterisation

#### 2.3.1. Physicochemical Analysis of Red Beetroot Peel Powder

The soluble solid content of the prepared RBPP was assessed by the refractometric method, using a Refractometer (Optika HR-150N, Ponteranica, Italy), and the results were expressed in °Brix according to OECD standards, 2018 [36,37,38]. Titratable acidity (also called total acidity) was determined by the titrimetric method. Samples were homogenised with distilled water and titrated with 0.1 N NaOH solution. The results were expressed in the prevailing acid, namely, % malic acid.

The total dry matter was evaluated by the oven-drying method at 105 ± 2 °C to constant mass [39,40].

#### 2.3.2. Bioactive Compound Extraction from Red Beetroot Peel Powder

For the extraction of the bioactive compounds, 1 g RBPP was mixed with 50 mL solvent (MeOH 50% acidified with 0.30% HCl) on a magnetic stirrer for 30 min at 45 °C. The resulting mixture was centrifuged for 30 min, at 5000 rpm and 4 °C (Hettich Zentrifugen Mikro 22R centrifuge, Tuttlingen, Germany) and the clear supernatant was collected, filtered through 0.45 µm filters and used for spectrophotometric determinations (total polyphenol content, antioxidant activity and total betalain content).

#### 2.3.3. Total Betalain Content of Red Beetroot Peel Powder

For the quantitation of betalain content, the spectrophotometric method previously described by Wruss et al. (2015) [41] and Lazăr (Mistrianu) et al. (2021) [42] was used. The content of betalains was calculated as the sum of the concentrations of betacyanins and betaxanthins. Briefly, the absorbance of the appropriately diluted extract was measured at 536 nm for betacyanins and at 485 nm for betaxanthins using T70 a UV-Vis spectrophotometer (PG Instruments Ltd., Alma Park, UK); then, the concentrations were calculated using the following equation:

(1) Betacyanins/Betaxanthins mg/L = (A × D × MW × 1000)/(ɛ × i)

where

betacyanins: A = absorbance read at 536 nm—absorbance read at 650 nm; MW (molecular weight) = 550 g/mol; ɛ = 60,000 (molar extinction coefficient in L× mol^−1^ × cm^−1^);

betaxanthins: A = absorbance read at 485 nm—absorbance read at 650 nm; MW (molecular weight) = 339 g/mol; ɛ = 48,000 (molar extinction coefficient in L× mol^−1^ × cm^−1^);

D: dilution factor;

i: path length (1 cm).

The results are presented as milligrams of betalains per 100 g of dry matter sample (mg betalains/100 g DM).

#### 2.3.4. Total Polyphenol Content of Red Beetroot Peel Powder

For the determination of total polyphenol content, the spectrophotometric Folin–Ciocâlteu method was used, following the protocol previously reported by Pereira et al. (2019) [43] with some modification. Briefly, 0.10 mL appropriately diluted extract was mixed with 7.90 mL distilled water, 0.50 mL of Folin–Ciocalteu reagent and 1.50 mL Na_2_CO_3_ 20% solution. The samples were kept in the dark, for incubation, for one hour; then, the absorbance was registered at 760 nm against the blank solution. The results were calculated based on the standard curve obtained with gallic acid as reference and are expressed as mg gallic acid equivalents per 100 g of dry matter sample (mg GAE/100 g DM).

#### 2.3.5. Antioxidant Activity of Red Beetroot Peel Powder

The analysis of the antioxidant activity by the DPPH method was performed according to the protocol previously described by Sielicka et al. (2014) [44] as follows: 0.10 mL of appropriately diluted extract (with the same solvent used for extraction) was mixed with 2.40 mL of DPPH (2,2-diphenyl-1-pikrylhydrazyl) solution of 2.50 mg/100 mL concentration. After incubation for 10 min, the absorbance was read at 515 nm. The results were calculated based on the standard curve obtained with Trolox and the results are presented as micromoles Trolox equivalent per gram of dry matter sample (µmol Trolox eq/g DM).

#### 2.3.6. Colourimetric Analysis of the Red Beetroot Peel Powder

The colourimeter MiniScan XE Plus, model 45/0-L (Hunter Associates Laboratory Inc., Reston, VA, USA) was used for the colourimetric analysis of RBPP. The parameters L*, brightness (+)/darkness (−); a*, red (+)/green (−) colour components; and b*, yellow (+)/blue (−) colour components were furnished by the device, while the parameters c*, chroma; h*, the hue angle; and ∆E*, the overall colourimetric difference were calculated with the following equations [45]:

(2) c* = (a*^2^ + b*^2^)^1/2^

(3) h* = arctan(b*/a*)

(4) ∆E* = (∆L^2^ + ∆a^2^ + ∆b^2^)^1/2^

### 2.4. Preparation of Dried Apple Snack

**Figure 2:**
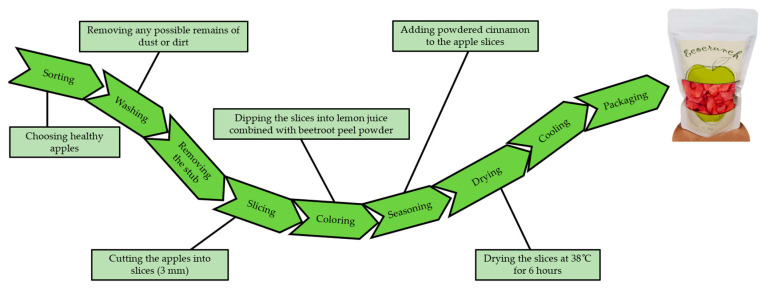
Experimental protocol for obtaining the dehydrated apple snack.

The enriched dried apple snack was prepared following the protocol shown in Figure 2.

The selected healthy apples were washed, cored and then sliced (3 mm thickness). In the next step, the apple slices were soaked in the RBPP mixture, seasoned with cinnamon (22 mg for 1 kg of fresh apple slices) and then dried using a basic hot-air dehydration device (Biovita Deluxe-10, Cluj-Napoca, Romania). To preserve the temperature-sensitive bioactive components, the apple slices were dried at 38 °C for six hours. After cooling, the samples were transferred to a zippered packaging and maintained at a controlled temperature of 10–12 °C.

Four different treatments were applied to enrich the dehydrated apple snack, as shown in Table 1, to which a control sample consisting of untreated apple slices, subjected to the same heat treatment, was added. Each apple snack sample was prepared in triplicate, in 1 kg batches, under laboratory conditions. Preliminary tests were carried out to determine the doses of beetroot peel powder, and for this investigation, samples of 5 and 10% RBPP were used, which proved to be the most appropriate following sensory analysis. These levels were selected to maintain the acceptable sensory qualities of the finished product while achieving notable nutritional benefits (antioxidants, polyphenols and betalains). Higher doses could have a negative impact on taste, colour intensity or texture, according to preliminary sensory evaluations, while lower amounts would not significantly improve nutritional value. For possible industrial use, the selected doses thus constitute an acceptable and practicable range.

### 2.5. Analysis of Dried Apple Snack

#### 2.5.1. Physicochemical Characterisation

The total soluble solids, total dry matter and treatable acidity were assessed as described for RBPP in Section 2.3.1. The pH was measured by the potentiometric method with a laboratory pH-meter (Testo 206-pH2, Lenzkirch, Germany), with the results being expressed in units of pH [46]. The ash content was determined according to the protocol described by Kocabıyık and Alkan (2025) [47] as follows: approximately 5 g of dry samples were carbonised in the calcination oven Snol (Telecomed, Iași, Romania) at 550 °C until a white ash with a constant mass is obtained. Schoorl’s method was used for reducing sugar determination [39]. Ascorbic acid (vitamin C) content was determined with a Reflectoquant (Lab Equipment, Merck, Darmstadt, Germany), a dispositive that measures light reflected from the test strip. The determination range is between 25 and 450 mg/L ascorbic acid and the results are expressed in mg/100 g of fresh product [46].

#### 2.5.2. Extraction Procedure for Spectrophotometric and HPLC Determinations of Apple Snack

The grinded dehydrated apple snack was extracted with the solvent (MeOH 50% acidified with 0.3% HCl) at a 1:10 (*w*/*v*) ratio, on a magnetic stirrer for 30 min, at 45 °C. The extract was centrifuged for 30 min, at 5000 rpm and 4 °C (Hettich Zentrifugen Mikro 22R centrifuge, Tuttlingen, Germany) and the clear supernatant was collected, filtered through 0.45 µm filters and used for spectrophotometric analysis and individual polyphenol determination by HPLC.

#### 2.5.3. Total Betalain Content, Total Polyphenolic Content and Antioxidant Capacity

The total betalain content, total polyphenolic content and antioxidant capacity were assessed according to the methodology presented in Section 2.3.3, Section 2.3.4 and Section 2.3.5, respectively.

#### 2.5.4. HPLC Analysis of Phenolic Compounds

Identification and quantification of the phenolic compounds from extracted samples were achieved as previously reported by Filimon et al. (2023) [48] with some minor modification. Briefly, the analyses were performed on a Waters 2695e Alliance HPLC system(Milford, Massachusetts, USA) coupled with a 2998 PDA Detector, and the separation of the phenolic compound was realised on a Waters XBridge C18 column (50 × 4.6 mm, 3.5 µm), maintained at 30 °C while monitoring a 280 nm wavelength. A binary mobile phase consisting of a solution of 0.1% TFA in water (mobile phase A) and a solution of 0.1% TFA in acetonitrile (mobile phase B) were used, at a flow rate of 0.7 mL/min. Quantification was realised based on an external standard curve for each individual phenolic compound.

### 2.6. Colourimetric Analysis of the Apple Snack

The analysis was carried out according to the methodology presented in Section 2.3.6.

### 2.7. Sensory Characteristics

Sensory evaluation was performed by a panel of 60 untrained consumers (30 men and 30 women of 20 to 60 years old). Members were chosen to identify the level of acceptance of dehydrated apple snacks. Using a 0–10 point hedonic scale (Table 2), panel members rated the product’s appearance, colour, smell, taste and consistency (1, strongly dislike; 10, strongly like), as described by Lawless and Heyman (2010) [49]. Samples were coded as control, S1, S2, S3 and S4 and served to the panel participants at around 11 am. Tasters were instructed to evaluate external appearance, colour, overall aroma, consistency, sweet taste, sour taste, flavour and acceptability, indicating their degree of liking or disliking the snacks by assigning a number, as provided in the hedonic scale, according to their preferences. Testing was completed in one session and each consumer rated all 5 samples. The samples were coded with different numbers from 1 to 5, and the order was changed, being randomly distributed, especially to obtain accurate results for sensory analysis. In sensory science, randomisation ensures that panellists do not develop order-based preferences or fatigue effects, while counterbalancing the serving order across panellists reduces positional bias. Untrained panellists were selected because the objective of this sensory evaluation was to assess consumer acceptability rather than detailed descriptive profiling. Untrained consumers are more reflective of real-world product users and are suited for hedonic testing. In this context, where apple chips are enhanced with functional ingredients like lemon juice (for browning prevention and acidity) and beetroot peel powder (for colour and added nutrients), the focus is on their overall liking and purchase intent of the snacks rather than nuanced sensory attributes. Hence, using untrained panellists aligns with the goal of measuring general consumer preferences.

Mentimeter software was used to collect real-time feedback from panellists involved in the sensory evaluation.

### 2.8. Statistical Test

The experimental data were statistically analysed using analysis of variance (ANOVA) to assess the significance of differences among the studied variants. Tukey’s honestly significant difference (HSD) test was used for mean separation at a significance level of 0.05, applied only to variables showing significant differences in ANOVA. All statistical analyses were performed using SPSS software version 21.0 (IBM Corp., Armonk, NY, USA). Results are presented as means ± standard errors.

## 3. Results and Discussion

### 3.1. Red Beetroot Peel Powder Characterisation

Prior to its use to enrich the apple snack, RBPP was analysed for its physicochemical characteristics and colour properties. The results are summarised in Table 3.

The chemical composition of beetroot peel can be correlated with the degree of ripening, the red beetroot variety and the dehydration process or temperature. Thus, during dehydration, water is removed, and soluble dry substances are concentrated [50]. The measurement expressed in degrees Brix (°Bx) shows the amount of soluble solids, mostly sugars, in the analysed powder. A high amount of 85.50 °Bx indicates a significant quantity of sugars and other soluble components that improves the powder’s overall sweetness and strengthens its impact on food products’ sensorial attributes and texture. A very low moisture content is shown by the powder’s exceedingly high TDM value of 98.41%. This prolongs its shelf life and prevents microbial growth, which is crucial for its preservation. In terms of pH, beetroot peel powder has a slight acidity with a pH value of 5.4. The powder’s total acidity, presented as a percentage of malic acid, indicates its overall acid content. A percentage of 1.32% signifies a detectable level of acidity, that could affect the powder’s ability to perform its functions (for example, as a flavour enhancer or preservative) and contribute to the flavour profile (sourness).

Analyses carried out on RBPP emphasised its rich content in bioactive compounds. For the total betalain content, a value of 1361.30 ± 2.45 mg/100 g DM was obtained. This is consistent with the results obtained by Sawicki et al. (2016) [51] for different beetroot varieties which ranged between 1026 and 1715 mg betalain/100 g DM. The authors also observed that the peel is the part of the root with the highest betalain content [51]. Regarding the total polyphenol content of the RBPP, a value of 2780.01 ± 68.38 mg GAE/100 g DM was obtained. A similar but slightly lower value of 2404 mg GAE/100 g DM was obtained in another study by Salamatullah et al. (2021) [52], when RBPP was subjected to extraction with 50% methanol at a temperature of 30 °C. The high content of betalains and polyphenols of RBPP is reflected by its antioxidant capacity of 503.96 ± 1.83 µmol TE/g DM, which is consistent with the fact that beetroot is considered among the ten most powerful vegetables in terms of antioxidant capacity [53].

Beetroot-derived pigments are available as the food additive E162 in the USA and Europe. The measured colour parameters of the RBPP showed a lightness of 28.09 ± 0.27, red shades indicated by the positive values of the a* parameter (34.74 ± 0.42) and yellow shades indicated by the positive values of the b* parameter (6.36 ± 0.03). The red and yellow shades can be correlated with the total betalain content that represents the sum of betacyanins and betaxanthins, which are classes of pigments responsible for red and, respectively, yellow colours [54]. The amount of betalains, which are nitrogenous water-soluble pigments, in beetroot varies depending on the cultivar, root section (inner part or peel), growth environment and processing techniques [55]. Bahriye et al. (2023) [56] showed that rising the beetroot drying temperature from 50 °C to 70 °C led to a decreasing trend in redness (a*) and yellowing (b*), while for lightness (L), an increasing trend was observed. Consequently, in our study, a lower drying temperature of 38 °C determined slightly higher values for a* and b* parameters and lower for the L parameter than reported by Bahriye et al. (2023) [56].

### 3.2. Apple Snack Characterisation

To produce a nutritious snack, fortified with bioactive components and visually appealing to customers, apple slices were immersed in mixtures with varying concentrations of RBPP during the manufacturing process (Figure 2). Along with apples and red beetroot peel, the proposed apple snack also contains lemon juice that was added to samples S3 and S4 in order to examine its effects. The biological activity of lemon (*Citrus limon*) is largely attributed to its substantial phenolic compound content, encompassing flavonoids such as diosmin, hesperidin and limocitrin, as well as phenolic acids like ferulic and *p*-hydroxybenzoic acids [57]. Furthermore, the essential oil of lemon is abundant in bioactive monoterpenoids, including D-limonene, β-pinene and γ-terpinene. Scientific investigations realised by Klimek-Szczykutowic et al. (2019) [58] have substantiated the therapeutic potential of *C. limon*, demonstrating anti-inflammatory, antimicrobial, anti-cancer and antiparasitic properties. Additionally, the potent antibacterial activity and the presence of citric acid, ascorbic acid, minerals and flavonoids within lemon essential oils [59] suggest its utility as a food preservative against foodborne pathogens.

Cinnamon, a spice that complements apples, was used to season the samples for enhanced flavour. The composition of cinnamon, i.e., *Cinnamomum zeylanicum* and *Cinnamomum cassia*, features essential oils and key derivatives such as cinnamaldehyde, cinnamic acid and cinnamate. Beyond its established use in the aroma and essence industries, where its fragrance is leveraged in food, perfume and medicinal products, cinnamon demonstrates significant biological activities [60]. These include antioxidant, anti-inflammatory, anti-diabetic, antimicrobial, anti-carcinogenic, lipid-lowering and cardiovascular disease-reducing effects [61].

In order to avoid the degradation of colour compounds and preserve nutritional values of the final product, the dehydration process was conducted at 38 °C, which represents a lower temperature compared with other studies [62,63].

**Figure 3:**
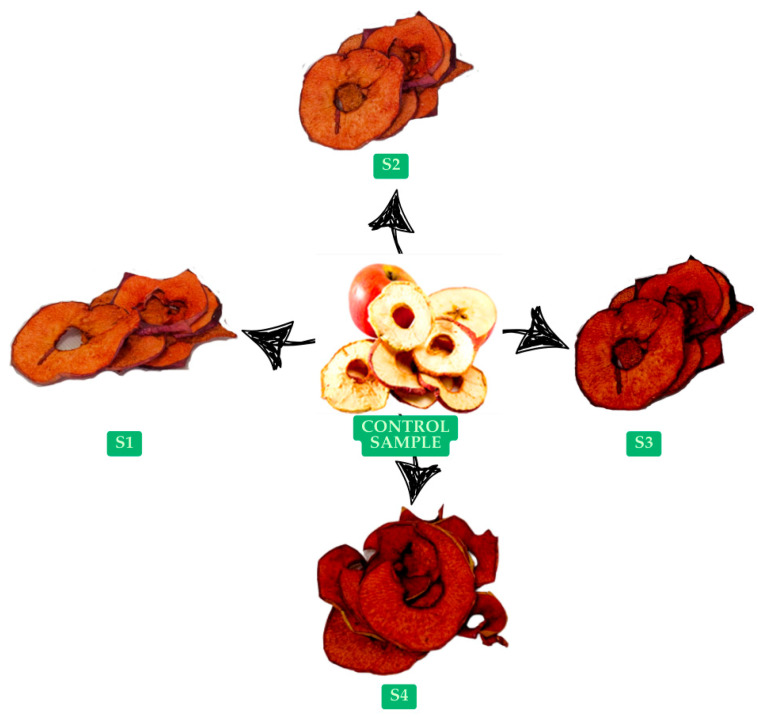
The experimental formulation of dried apple snacks. Control sample; S1 = apple slices dipped in 5% RBPP in water; S2 = apple slices dipped in 10% RBPP in water; S3 = apple slices dipped in 5% RBPP in 50% lemon juice; S4 = apple slices dipped in 10% RBPP in 50% lemon juice; all formulations are seasoned with cinnamon powder.

Figure 3 shows the four enriched samples obtained and the untreated sample that was prepared as a control.

#### 3.2.1. Physicochemical Characteristics of Dehydrated Apple Snacks

In Table 4, the main parameters of apple snacks are presented: total soluble solids (TSS), total acidity (TA), pH (units of pH), moisture (%), total dry matter (TDM), ash (%) and ascorbic acid content (mg/100 g product).

Numerous processing and formulation factors affect the dried apple snacks’ physicochemical properties. Temperature and drying techniques are crucial. While longer drying periods and higher temperatures can lower water activity and moisture content, they can also lower the total phenolics and affect texture, bulk density, porosity and colour through enzymatic browning or Maillard reactions. Second, pH is lowered by the addition of acidic substances (lemon juice), which aids in blocking polyphenol oxidase and maintaining phenolic stability and colour. Additionally, adding beetroot peel powder to a formulation provides dietary fibre, polyphenols and pigments (betalains), which alters the product’s ability to bind water, its viscosity and its antioxidant potential. Lastly, water activity, crispness, shelf life stability and bioactive retention can all be impacted by storage conditions, particularly temperature, relative humidity and packing environment. The final product’s moisture content, texture, colour, density and antioxidant quality are all determined by these characteristics combined simultaneously.

The soluble solid content is primarily composed of sugars; an index correlated with the maturity of fruits [46]. The control sample had the lowest soluble solid concentration (27.4%), whereas sample S4 had the highest (49.50%). The increases in soluble solids in samples S1–S4 are significant compared to the control sample.

Fruit quality changes are frequently indicated by the degree of acidity (pH). The level of acidity of fruits depends on fruit, variety and ripeness, but typically have a low pH value [23,64]. The pH values range from 3.48 to 4.13, indicating a slight variation in acidity. The control sample has a pH of 3.80, while S2 and S1 show the highest values (4.13 and 4.07, respectively), with significant differences from the other samples, indicating that these vegetable-added formulations can reduce acidity and give the product a less sour taste.

Acidity represents a key factor correlated with the fruit’s quality and freshness. The major organic acid in mature apple fruit is malic acid [65]. Sample S4 registered the higher acidity value (2.47%), while the control (B) has an acidity of 0.45%. The increased acidity in S1–S4 indicates that additional organic acids are contributed by other products, such as lemon juice, which could improve the sensory qualities by counteracting sweetness. According to Owusu et al. (2012) [66], the total acidity values of the dried apple snacks increased due to the organic acids in apples that become more concentrated [66]. But, in different experimental conditions, at higher temperatures, the organic acids may dissociate faster, which could explain the samples’ declining pH and titratable acidity levels. Similar results were also confirmed by Ghinea et al. (2022) [32] and Kahraman et al. (2021) [28].

One crucial aspect of fruit and vegetable quality that has a direct impact on their stability is the moisture content [67]. The moisture content, which ranges from 12.27% to 12.63%, is rather constant. The results show that all samples have been effectively dehydrated, guaranteeing product stability and shelf life.

Drying process develops changes in the dry matter contents and drastically lowers the moisture content of dietary components. As samples dry, their moisture content varies, which can lead to the release of organic and volatile organic compounds, the breakdown of pigments and modifications in their chemical structure, according to Mongi et al. (2015) [68]. For the values obtained for the dry matter, no statistically significant difference was observed (87.37–87.73%) among the five samples analysed, confirming the effectiveness and constancy of the dehydration process.

Ash is the inorganic matter resulting after any sample mineralisation, including food samples. The compounds contained in the food sample and the mineralisation technique determine the ash composition [69]. In the control sample, the ash content (which reflects the mineral content of the sample) was 0.46%; in S4, it increased to 0.78%. The increasing amount of ash indicates that adding vegetable by-products improves the dehydrated apples’ mineral composition and may increase their nutritional value.

Generally, the reducing sugar content of the dried fruits in net weight was found to be higher than the content of fresh fruits. This is due to moisture loss [70]. A significant increase in reducing sugars (glucose) was observed in the treated samples, with S4 showing the highest value (28.40 ± 0.15%).

Vitamin C levels for each sample have either been similar to or different from findings previously reported by other authors [71]. These variations may be due to factors such as fruit cultivar, maturity stage and methods used for vitamin C extraction and determination [71]. In terms of ascorbic acid content, it can be observed that the control sample (B) showed the lowest levels of ascorbic acid at 3.87 ± 0.15 mg/100 g of product, followed by S1 (6.45 ± 0.17), S2 (8.26 ± 0.19) and S3 (35.08 ± 0.12). The highest concentrations were found in sample S4, which showed increased values of 39.19 ± 0.16 mg/100 g of the product.

#### 3.2.2. Bioactive Compound Content in Apple Snacks

The prepared dehydrated apple snacks were evaluated for their content of bioactive compounds (total betalain content and total polyphenolic content—TPC) and antioxidant activity. The results presented in Table 5 show that the use of red beetroot peel enriched the apple snacks with betalains, increased the content in polyphenols and improved the antioxidant activity.

The total betalain content of the treated dehydrated apples increased with the amount of red beetroot peel used in the treatment mixture and ranged between 22.83 ± 1.29 and 67.01 ± 4.18 mg betalains/100 g DM. It can be observed that the samples that include lemon juice have a significantly higher total betalain content than samples with the same amount of red beetroot but without lemon juice. This fact is consistent with the stability of betalains in slightly acidic conditions, thus facilitating their use in acidic food [72].

The increase in total polyphenolic compounds of apple snacks with RBPP was significant: from 373.11 ± 32.09 mg GAE/100 g DM for the control, to 903.22 ± 28.95 mg GAE/100 g DM for sample S4, prepared with the highest amount of RBPP and lemon juice. The use of lemon juice positively influenced the total polyphenol content, as samples prepared with lemon juice (S3 and S4) recorded higher values than those without (S1 and S2). This fact can be explained by the lower pH value of samples prepared with lemon juice, which reduces the polyphenol oxidase activity and prevents the degradation of polyphenol [73,74]. Citric acid and sodium bisulphite are added in order to prevent apples from undergoing enzymatic browning. The degree of browning depends on the phenolic compound content and the polyphenol oxidase activity. Citric acid produces a pH reduction, thus decreasing the enzyme activity, whereas sodium bisulphite is the most potent polyphenol oxidase inhibitor in apples [73].

Betalains and phenolic compounds are bioactive compounds that have a variety of health benefits mainly associated with their antioxidant properties [75,76]. A molecule that has the ability to protect other molecules from oxidising is called an antioxidant. Research on the significance of plant-derived antioxidants in food and human health has gained attention lately [77]. When found in food, even in very small amounts, antioxidants delay, control or prevent oxidative processes that affect food quality. Also, the antioxidants are associated with beneficial health proprieties, preventing the initiation and propagation of degenerative diseases in the human body [78]. Thus, in the current study, the antioxidant activity of the five prepared dehydrated apple snacks was evaluated based on the DPPH assay. It can be observed that treated apples registered significantly higher antioxidant activity compared to the untreated control. The improvement in antioxidant activity can be attributed to bioactive compounds from red beetroot peel (polyphenols, flavonoids, carotenoids, betalains, etc.). Vitamin C, which is found in higher amounts in the peel than in other parts of beetroot [79], can also contribute to improving the antioxidant capacity of the enriched product [80]. The highest values for antioxidant potential were registered for samples S2 (36.61 ± 0.29 mmol TE/g DM) and S4 (37.11 ± 0.54 mmol TE/g DM), i.e., those prepared with double the amount of red beetroot peel powder compared to the amount used for samples S1 and S3. The slight difference between samples S2 and S4 is probably due to the lemon juice used in the preparation of sample S4, which has also been described as an antioxidant [81]. The cinnamon added to the recipe, in addition to enhancing the flavour of the product, also led to improved antioxidant activity, which is consistent with that previously observed by Tarko et al. (2010) [63] and (2023) [82] in studies involving apple snacks prepared with this spice. Demiray investigated the influence of temperature used for the preparation of apple snacks on the antioxidant activity and concluded that a temperature of 45 °C (the thickness 1.50 mm) is favourable, while higher temperatures were disadvantageous [62].

The peel of red beetroot was used for the fortification of other food products with the effect of increasing the content of bioactive compounds and antioxidant activity. The meringue with red beetroot peel prepared by Constantin et al. (2025) [83], in addition to the pleasant colour, had higher total polyphenolic content and antioxidant activity compared to the control meringue without red beetroot. The same effect was observed by Lazar et al. (2022) [84] when they used red beetroot peel to prepare value-added mayonnaise.

### 3.3. HPLC Polyphenolic Profile of Apple Snacks

**Figure 4:**
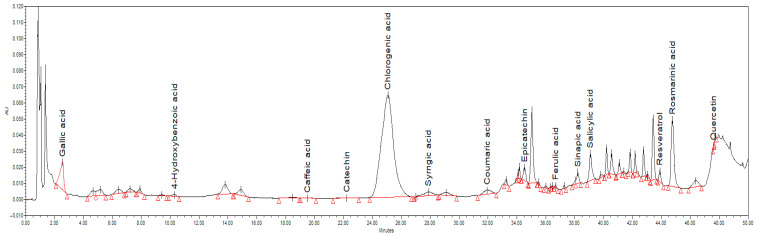
HPLC-DAD chromatogram at 280 nm obtained for sample S4.

Apple fruits contain a high concentration of phenolics, which provide numerous health benefits due to their antioxidant and anti-inflammatory activity [85]. Phenolic compounds in apples range from low-molecular-weight structures to complex molecules such as tannins and polyphenol derivatives [86]. The content of individual phenolic compounds depends considerably on the variety, maturity, storage and growing conditions of the apple [85]. In this study, based on the HPLC method, 14 individual phenolic compounds belonging to the classes of phenolic acids (gallic acid, 4-hydroxybenzoic acid, caffeic acid, chlorogenic acid, syringic acid, ferulic acid, coumaric acid, sinapic acid, salicylic acid and rosmarinic acid), stilbenes (resveratrol) and flavonoids (catechin, epicatechin and quercetin) were identified (Figure 4).

The concentration of each identified phenolic compound (µg/g DM) in the prepared apple snack is shown in Table 6.

Chlorogenic acid was found to be the major phenolic compound in the analysed samples. The concentrations measured in the control and the four tested samples showed no statistically significant differences, ranging from 690.53 to 705.34 µg/g DM. This indicates that the applied treatments did not affect the chlorogenic acid content, suggesting that its presence in the analysed apple snacks originates primarily from the apple itself.

Tarko et al. (2010) [63] also found chlorogenic acid as the major phenolic in flavoured apple chips, ranging from 610.80 to 762.80 µg/g DM. Other previous research [87,88] have shown chlorogenic acid as the predominant phenolic compound in certain apple cultivars. Furthermore, of the seven apple cultivars examined, Cvetković et al. (2024) [87] found that Idared (the variety used in the current study) had the greatest concentration of chlorogenic acid. Another study showed that cultivated apples have higher chlorogenic acid contents compared to wild apples [89]. Given its antioxidant and anti-inflammatory properties [90], chlorogenic acid is one of the compounds that significantly contributes to the nutraceutical value of apples and apple products.

After chlorogenic acid, rosmarinic acid (92.13–112.52 µg/g DM) and salicylic acid (72.14–117.09 µg/g DM) were found in the highest amounts in the apple snacks. The origin of these two phenolic acids in the apple snacks is the apple, since there are limited data on their endogenous presence in red beetroot or lemon juice. However, in the samples treated with lemon juice, S3 and S4 showed slightly higher amounts. Rosmarinic acid was also identified in the apple skin of a different cultivar (3.02–3.74% of total dry skin) by Amzad Hossain et al. (2009) [91], while Lee et al. (2017) [92] identified it in the peel (3.20–34.10 µg/g DM) and pulp (0.40–2.60 µg/g DM). The higher amounts of rosmarinic acid found in our study compared to prior research may be attributed to the thermal processing. Ugurulu and Bakkalbasi (2024) [86] reported a higher amount of some phenolic compound in apple snacks than in fresh apples, which was explained by the release of bounded phenolics during thermal treatment. Soares et al. (2008) [93] found salicylic acid as the predominant free phenolic acid in apples from Gala and Fuji cultivars. In addition, other studies [45,94] have observed the presence, in significant quantities, of this phenolic in apple pomace.

Important amounts of epicatechin were registered in the analysed samples. The treatment used in sample preparation strongly influenced its content, that was about two-fold higher in sample S1 (24.41 µg/g DM), three-fold higher in samples S2 (28.97 µg/g DM) and S3 (31.15 µg/g DM) and four-fold higher in sample S4 (41.61 µg/g DM), compared to the control sample (10.13 µg/g DM). Liaudanskas et al. (2014) [95] showed that the content of this flavonoid in apple fruits varies significantly between cultivars. Also, the different drying methods applied to obtain apple snacks led to different amounts of epicatechin (34.33–137.07 µg/g DM) [86]. As expected, the rise in the concentration of this flavonoid in the flavoured apple snack samples S1–S4 was consistent with the amount of RBPP used in the preparation, with the presence of epicatechin in red beetroot being previous reported [96,97,98]. In addition, Tarko et al. (2010) [63] showed that cinnamon led to an increased content of epicatechin when used to prepare apple chips. The stabilising effect of lemon juice can also be observed for epicatechin, given that the samples with lemon juice (S3 and S4) recorded higher concentrations than those prepared with the same amount of RBPP but without lemon juice (S1 and S2).

Catechin is another flavonoid found in the prepared apple snack, but in small amounts. However, its content augmented with the amount of RBPP used in the recipe and when lemon juice was included. The apple snack prepared by Ertekin Filiz and Seydim (2018) [99], Tarko et al. (2010) [63], Uğurlu and Bakkalbaşı et al. (2024) [86] also contained catechin, while Abdo et al. (2020) [100] found in the peel of red beetroot a concentration of 184.50 µg catechin/g DM. A similar evolution was observed for quercetin, a flavonoid that was previously noticed in both apple snack [101] and red beetroot peel [96].

Regarding syringic acid, an amount of 12.57 µg/g DM was determined for the control apple snacks. This phenolic is found in apple fruits of different varieties [102], but its levels decrease as the fruit approaches harvest [103]. For the treated apple snacks, an increased amount of syringic acid was obtained (17.15–18.54 µg/g DM) due to the addition of this phenolic acid, which is endogenously found in red beetroot [104].

Ferulic acid and coumaric acid were found in the prepared apple snack in low amounts of 0.80–1.33 µg/g DM and 5.26–6.39 µg/g DM, respectively. Both phenolic acids were previously identified in Idared fresh apple [105] and dehydrated red beetroot [104].

Low amounts of resveratrol were registered in the prepared apple snacks, ranging from 3.61 to 5.07 µg/g DM. According to Geană et al. (2021) [105], the fresh apples of the Idared variety presented the lowest concentration of resveratrol (12.50 µg/g DM) among the 14 analysed variates. Small amounts of resveratrol (25.50 µg/g DM) were also reported for red beetroot dehydrated in a microwave [104]. Thus, the apple snack prepared with RBPP showed slightly higher resveratrol content than the control apple snack, but was not significantly statistically different.

Gallic acid, *p*-hydroxybenzoic acid, caffeic acid and sinapic acid were the phenolic acids found only in the treated samples (S1–S4). According to Carrillo et al. (2019) [106], gallic acid is the predominant phenolic acid in red beetroot, and its level is maintained high in the dehydrated red beetroot as well [104]. Consequently, in our study, sample S4, which was prepared with double the quantity of RBPP and lemon juice, had the highest concentration of gallic acid (32.79 µg/g DM), with a difference compared to samples S1–S3 that is statistically significant. Low levels of *p*-hydroxybenzoic acid, caffeic acid and sinapic acid were reported by Płatosz et al. (2020) [107] in fresh red beetroot.

### 3.4. Colourimetric Analysis of Dehydrated Apple Snack

Food colouring significantly influences the appeal and consumer acceptance of many food and beverage products. While augmenting the range of colours in food may promote increased consumption, the visual perception of some hues, particularly those deemed unappealing, can inhibit appetitive behaviours. Concerns have been consistently raised about alleged negative health effects related to the consumption of specific artificial food dyes, which has led food manufacturers to search for natural dyes that appeal to consumers [103]. Thus, in the current study, the effect of RBPP on the colour of the apple snacks was evaluated, and the results are shown in Table 7.

Compared to the control sample, the treated samples S1–S4 registered significant increased redness and yellowness as reflected by the positive values of parameters a* and b*, respectively. This increase is consistent with the total betalain content of the samples. It has previously been shown that red shades of food are associated with freshness and can improve the consumer’s appetite [108,109,110]. Conversely, the brightness parameter L significantly decreases as a result of the applied treatments. The rise in the pigment concentration is correlated with the colour intensification and the decrease in lightness (L*), as can be observed in this study for sample 4 in particular. Similar results were also obtained by Zhu et al. (2022) [111].

The ΔE* value indicates the overall colourimetric difference between two samples. Colour variations are classified as minor if ΔE* < 1.5, distinct if 1.5 < ΔE* < 3, and very distinct if ΔE* > 3 [112]. The overall colourimetric difference between the control sample and the treated samples was very distinct, as shown by the high values recorded, which significantly increased with the amount of RBPP used, from 20.28 for S1 to 45.07 for S4.

Over time, the colourimetric analysis of red beet by-products has been increasingly used due to the positive impact of its main pigments (betanin and betalain) for human health, which has been confirmed by Stoica et al. (2025) [12] and Azeredo (2009) [72]. The increased redness (a*) resulted in enhancing the visual aspect, which is in line with findings by González-Montelongo et al. (2010) [113], who demonstrated that natural colourants from fruit and vegetable by-products can improve the chromatic attributes of food products. Similarly, Sogi et al. (2015) [114] highlighted the stabilising effect of natural pigments like betalains during low-temperature drying.

### 3.5. Sensory Analysis

Food product sensory qualities are assessed through sensory evaluation, a technique that relies on human perception. This involves consumer panels, which measure product liking, or trained panels, whose expertise is developed and maintained through standardised training and performance evaluation.

The intensity of the major sensory characteristics of the dried products was generally greatly impacted by the drying techniques [115], the pre-treatments and by the raw materials. Table 8 lists the findings of the sensory evaluation of the final products analysed in this study. The samples were scored for characteristics such as external appearance, colour, overall aroma, consistency, sweet taste, sour taste, flavour and acceptability. The means and standard deviation are presented and discussed.

The physicochemical features of dried apple chips have a direct impact on their sensory qualities, including consistency, sweetness, colour and general acceptance. Texture is greatly influenced by moisture content; reduced moisture content tends to improve crispness and crunchiness, which are favourably correlated with sensory appeal. A brighter and more natural apple colour (higher L* and b*) is generally chosen. Similarly, colour factors (e.g., L, a*, b*) influence visual acceptance. Key components of flavour perception are perceived sweetness and tartness, which are influenced by total soluble solids and acidity. Further influencing customer taste is the retention or development of volatile chemicals during dehydration, which can either improve or lessen the apple’s flavour and scent. Consequently, enhanced sensory quality and customer satisfaction might result from processing that optimises physicochemical characteristics.

Simple dehydrated apples are already known on the market, but they are not as popular. Soluble solid content is a good indicator of the sugar content of apples and presumably of sweetness [116]. In this study, the samples evaluated with the highest sweet taste score (S1 and S2 (9.60)) also presented higher values of total soluble solid content compared to the control sample. However, samples S3 and S4 recorded a higher total soluble solid content than S1 and S2 but obtained a lower sweet taste score. This fact can be explained by the use of lemon juice, which balances the sweet taste.

The acidity of a food product could be a useful indicator of taste [116]. This parameter could be useful in assessing the acceptability of dried fruits, as consumers show clear preferences for apples with sweet or acidic taste; these results were also confirmed by Skendrović Babojelić et al. (2007) [117] and Antal et al. (2015) [118]. In agreement with these literature findings, sample S4, which has the highest acidity in the current study, was evaluated by the panellists as having a higher score for sour taste (9.50).

In terms of flavour, it can be observed that the values range from 6.80 ± 0.17 (control) to 9.90 ± 0.19 (S3). The most appreciated sample was S3, followed by S4.

In addition to nutritionally enriching the dehydrated apples, the red beetroot peel gives the apple snack an attractive red colour, which was highly appreciated by the evaluators; thus, sample S4 obtained the best score at 9.90.

The highest acceptability was registered for sample S3 with the value of 9.80 ± 0.15, followed by S4 (9.20 ± 0.10). The lowest value from this point of view was for the control sample 7.80 ± 0.10.

Overall, the enriched apple snack (S1–S4) achieved a significantly higher score compared to the control sample, for all sensory parameters evaluated, with the best score for samples that had lemon juice included in their recipes (S3 and S4). Taking into account the results of the sensory evaluation, the snack can be considered as having a balanced sweet–sour taste, simultaneously satisfying the concerns of modern consumers who desire a healthy lifestyle. In general, the attractive colour and the balanced sweet–sour taste were key descriptors of the resulting product, obtained from natural raw materials. Similarly, juices enriched with beetroot peel powder reported by Abdo et al. (2022) [119] were highly rated by evaluators for colour and taste.

**Figure 5:**
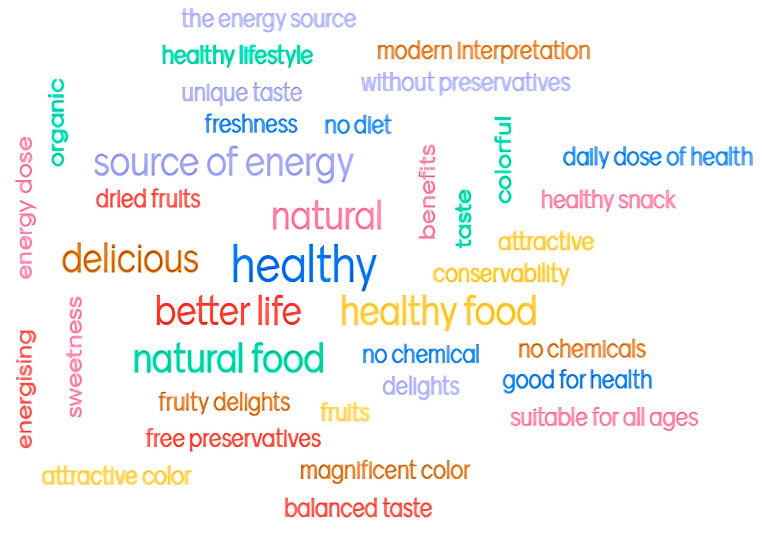
Consumers’ perceptions of the apple snack.

For the overall evaluation of the analysed apple snacks, the evaluators—using the mentimeter program—provided short descriptions of the samples, as shown in Figure 5. These descriptive insights are offered as exploratory findings.

In the mentimeter image, the largest words represent the responses from multiple people who used the same word. The smallest words represent responses from a smaller number of people. In this sense, the obtained apple snacks can be associated with the following terms: “source of energy”, “healthy”, “natural food”, “better life” and “delicious”, followed by “no chemicals”, “balanced taste”, “attractive colour”, “modern interpretation”, “without preservatives” and “suitable for all ages”, among others. These answers provide pertinent and realistic consumer viewpoints, although they are qualitative in character and unsupported by additional analyses.

## 4. Conclusions

The outcomes of this research support the hypothesis that red beetroot peel, a by-product of the agri-food industry that is rich in bioactive compounds and pigments, can be reintroduced in the food chain to obtain a value-added new food product, such as a dehydrated apple snack. The proposed apple snacks offer consumers a healthy food alternative, free of preservatives and synthetic dyes, without added sugar and low in calories. The investigation emphasised an important increase in bioactive compound content (betalains, polyphenols), antioxidant activity and consumer acceptability for the enriched apple snacks, compared to the control. The enriched apple snacks can be an alternative for unhealthy snacks due to their biochemical composition, attractiveness (red colour due to red beetroot peel powder), and especially for their balanced taste, as seen in S3 and S4. The products are intended to target all consumer groups, but especially young people and children, as they are accessible, healthy, tasty, attractive (red colour) and have a high nutritional and functional value (betalains, antioxidant activity, etc.). The findings of this research underscore the importance of developing innovative, visually pleasing and palatable products with a balanced nutritional profile to enhance consumer acceptance. The product formulated in this study presents a viable alternative that aligns with these objectives and may contribute to the diversification of health-oriented food offerings. More investigations will be conducted to optimise the ratio between ingredients, the dehydration process (different temperatures) and other apple varieties. In addition to high nutritional and sensory qualities, the new food product is in agreement with the principles of sustainability through the reduction in food waste and better use of natural resources. The favourable results obtained in this study highlight the importance of dehydrated fruits as a concentrated and versatile nutrient source and open new opportunities to expand the protocol to other fruits and vegetables, which meet the modern consumer’s increasing interest in healthy, sustainably produced foods.

## Figures and Tables

**Table 1:** Applied treatments used for apple snack preparation.

Sample	Treatment Details
Control	Apple slices
S1	Apple slices dipped in 5% RBPP in water, seasoned with cinnamon powder
S2	Apple slices dipped in 10% RBPP in water, seasoned with cinnamon powder
S3	Apple slices dipped in 5% RBPP in 50% lemon juice, seasoned with cinnamon powder
S4	Apple slices dipped in 10% RBPP in 50% lemon juice, seasoned with cinnamon powder

**Table 2:** Hedonic scale used to rate the sensory characteristics of the dehydrated apple snack.

Attribute	0—Minimum	10—Maximum
**External appearance**	Non-attractive	Very attractive
**Colour**	Bright	Dark
**Overall aroma**	Unpleasant	Very pleasant
**Consistency**	Very hard	Soft
**Sweet taste**	Imperceptible	Very intense
**Sour taste**	Imperceptible	Very intense
**Flavour**	Uncharacteristic	Aromatic, palatable
**Acceptability**	Poor quality	Very good quality

**Table 3:** Physicochemical profile and colour parameters of red beetroot peel powder.

Parameter	Results
**pH**	5.40 ± 0.23
**Soluble dry matter (°Bx)**	85.50 ± 0.41
**Total dry matter (%)**	98.41 ± 0.11
**Total acidity (% malic acid)**	1.32 ± 0.07
**Total betalain content (mg betalains/100 g DM)**	1361.30 ± 2.45
**Total polyphenol content (mg GAE/100 g DM)**	2780.01 ± 68.38
**Antioxidant activity (µmol TE/g DM)**	503.96 ± 1.83
L*	28.09 ± 0.27
a*	34.74 ± 0.42
b*	6.36 ± 0.03
c*	35.32 ± 0.42
h*	10.38 ± 0.02

Note: Values are presented as the means of three determinations ± standard deviation.

**Table 4:** Physicochemical parameters of dehydrated apple snacks.

Sample	Control	S1	S2	S3	S4
**Total soluble solids (%)**	27.40 ± 0.01 ^e^	35.03 ± 0.03 ^d^	42.08 ± 0.02 ^b^	38.11 ± 0.04 ^c^	49.50 ± 0.03 ^a^
**pH**	3.80 ± 0.01 ^b^	4.07 ± 0.02 ^a^	4.13 ± 0.03 ^a^	3.48 ± 0.01 ^d^	3.60 ± 0.01 ^c^
**Total acidity ** **(% malic acid)**	0.75 ± 0.09 ^b^	0.56 ± 0.10 ^b^	0.63 ± 0.08 ^b^	2.13 ± 0.06 ^a^	2.47 ± 0.08 ^a^
**Moisture (%)**	12.27 ± 0.01 ^d^	12.36 ± 0.01 ^c^	12.56 ± 0.01 ^ab^	12.49 ± 0.03 ^b^	12.63 ± 0.01 ^a^
**Total dry matter (%)**	87.73 ± 0.18 ^ns^	87.64 ± 0.02 ^ns^	87.44 ± 0.03 ^ns^	87.51 ± 0.00 ^ns^	87.37 ± 0.03 ^ns^
**Ash (%)**	0.46 ± 0.01 ^d^	0.65 ± 0.00 ^c^	0.72 ± 0.01 ^b^	0.71 ± 0.00 ^b^	0.78 ± 0.01 ^a^
**Reducing sugars ** **(% Glucose)**	21.30 ± 0.03 ^e^	25.20 ± 0.01 ^d^	27.60 ± 0.00 ^b^	26.10 ± 0.02 ^c^	28.40 ± 0.02 ^a^
**Ascorbic acid ** **(mg/100 g product)**	3.87 ± 0.01 ^e^	6.45 ± 0.01 ^d^	8.26 ± 0.03 ^c^	35.08 ± 0.02 ^b^	39.20 ± 0.01 ^a^

Note: Values are presented as the mean of three determinations ± standard deviation. Within each row: ^ns^—no statistically significant difference; values associated with the same lowercase letters are not significantly different at *p* ≤ 0.05 according to Tukey’s test.

**Table 5:** Phytochemical profile of dehydrated apple snacks.

Parameter	Sample				
	Control	S1	S2	S3	S4
**Total betalain content (mg betalains/100 g DM)**	n.a. ^d^	22.83 ± 0.74 ^c^	34.56 ± 1.93 ^b^	41.20 ± 1.65 ^b^	65.01 ± 4.26 ^a^
**Total polyphenol content (mg GAE/100 g DM)**	373.11 ± 18.53 ^d^	497.68 ± 17.08 ^c^	601.33 ± 17.98 ^b^	544.51 ± 15.58 ^bc^	903.22 ± 16.71 ^a^
**Antioxidant activity ** **(µmol TE/g DM)**	28.07 ± 0.22 ^c^	33.30 ± 0.29 ^b^	35.51 ± 1.25 ^ab^	33.90 ± 0.16 ^b^	37.11 ± 0.31 ^a^

Note: mg GAE/100 g = milligrams of gallic acid equivalents per 100 g of dried sample; µmol TE/g DM = micromoles of Trolox equivalents per gram of dried sample. Values are presented as the mean of three determinations ± standard deviation; n.a.—not applicable. Within each row, values associated with the same lowercase letters are not significantly different at *p* ≤ 0.05 according to Tukey’s test.

**Table 6:** Results of HPLC quantification of individual phenolic compounds.

Phenolic Compound (µg/g DM)	Sample				
	Control	S1	S2	S3	S4
**Gallic acid**	n.d. ^c^	3.44 ± 0.16 ^c^	3.17 ± 0.11 ^c^	19.60 ± 0.97 ^b^	32.79 ± 1.41 ^a^
***p *** **-Hydroxybenzoic acid**	n.d. ^c^	2.08 ± 0.18 ^b^	2.71 ± 0.10 ^b^	2.12 ± 0.15 ^b^	4.70 ± 0.32 ^a^
**Caffeic acid**	n.d. ^c^	n.d. ^c^	n.d. ^c^	0.90 ± 0.01 ^a^	0.24 ± 0.01 ^b^
**Catechin**	1.24 ± 0.01 ^c^	4.86 ± 0.07 ^b^	5.84 ± 0.19 ^a^	4.76 ± 0.12 ^b^	6.06 ± 0.02 ^a^
**Chlorogenic acid**	690.53 ± 13.86 ^ns^	692.78 ± 13.11 ^ns^	705.34 ± 15.50 ^ns^	691.29 ± 18.15 ^ns^	697.63 ± 24.53 ^ns^
**Syringic acid**	12.57 ± 0.57 ^b^	17.67 ± 0.57 ^a^	18.14 ± 0.70 ^a^	17.15 ± 1.15 ^a^	18.54 ± 1.18 ^a^
**Coumaric acid**	6.39 ± 0.21 ^ns^	5.26 ± 0.38 ^ns^	5.35 ± 0.28 ^ns^	6.31 ± 0.31 ^ns^	5.97 ± 0.17 ^ns^
**Epicatechin**	10.13 ± 0.88 ^d^	24.41 ± 0.83 ^c^	28.97 ± 0.98 ^bc^	31.15 ± 1.09 ^b^	41.61 ± 1.37 ^a^
**Ferulic** **acid**	0.81 ± 0.03 ^c^	0.80 ± 0.03 ^c^	0.81 ± 0.03 ^c^	1.33 ± 0.07 ^a^	1.08 ± 0.05 ^b^
**Sinapic acid**	n.d. ^b^	2.54 ± 0.39 ^b^	3.48 ± 0.21 ^b^	16.31 ± 1.89 ^a^	21.17 ± 2.40 ^a^
**Salicylic acid**	72.14 ± 5.67 ^b^	76.36 ± 4.89 ^b^	75.15 ± 4.50 ^b^	118.02 ± 5.52 ^a^	117.09 ± 5.96 ^a^
**Resveratrol**	3.61 ± 0.25 ^ns^	4.34 ± 0.36 ^ns^	5.07 ± 0.41 ^ns^	3.93 ± 0.31 ^ns^	4.98 ± 0.50 ^ns^
**Rosmarinic acid**	92.13 ± 4.39 ^ns^	107.91 ± 4.84 ^ns^	108.56 ± 5.36 ^ns^	108.69 ± 5.95 ^ns^	112.52 ± 4.60 ^ns^
**Quercetin**	1.74 ± 0.17 ^b^	1.78 ± 0.16 ^b^	1.93 ± 0.18 ^b^	3.55 ± 0.36 ^a^	3.78 ± 0.29 ^a^

Note: Values are presented as the mean of three determinations ± standard deviation; n.d.—not detected. Within each row: ^ns^—no statistically significant difference; values associated with the same lowercase letters are not significantly different at *p* ≤ 0.05 according to Tukey’s test.

**Table 7:** Colour parameters of apple snacks.

Colour Parameter	Sample				
	Control	S1	S2	S3	S4
L*	76.62 ± 0.37 ^a^	58.37 ± 0.43 ^b^	50.10 ± 0.28 ^c^	40.82 ± 0.35 ^d^	35.02 ± 0.16 ^e^
a*	12.35 ± 0.07 ^c^	20.50 ± 0.18 ^b^	22.70 ± 0.10 ^ab^	25.53 ± 1.52 ^a^	25.53 ± 1.52 ^a^
b*	2.67 ± 0.05 ^d^	3.94 ± 0.04 ^c^	2.51 ± 0.08 ^d^	5.26 ± 0.12 ^b^	5.84 ± 0.10 ^a^
c*	12.63 ± 0.06 ^e^	20.87 ± 0.18 ^d^	23.15 ± 0.12 ^c^	27.70 ± 0.19 ^b^	29.68 ± 0.51 ^a^
h*	12.19 ± 0.24 ^b^	10.89 ± 0.09 ^c^	23.15 ± 0.12 ^a^	10.95 ± 0.16 ^c^	11.41 ± 0.12 ^c^
ΔE*	- ^e^	20.28 ± 0.37 ^d^	28.79 ± 0.16 ^c^	38.95 ± 0.34 ^b^	45.06 ± 0.04 ^a^

Note: Overall colourimetric difference was calculated for each enriched apple snack compared to control apple snacks. Values are presented as the mean of three determinations ± standard deviation. Within each row, values associated with the same lowercase letters are not significantly different at *p* ≤ 0.05 according to Tukey’s test.

**Table 8:** Sensory characteristics (score) of apple snack.

Sensory Parameter	Sample				
	Control	S1	S2	S3	S4
External appearance	8.50 ± 0.02 ^e^	9.20 ± 0.02 ^d^	9.35 ± 0.01 ^c^	9.60 ± 0.02 ^b^	9.90 ± 0.02 ^a^
Colour	8.90 ± 0.03 ^e^	9.20 ± 0.01 ^d^	9.40 ± 0.02 ^c^	9.70 ± 0.02 ^b^	9.90 ± 0.01 ^a^
Overall aroma	7.00 ± 0.10 ^d^	8.70 ± 0.05 ^c^	8.95 ± 0.02 ^b^	9.70 ± 0.01 ^a^	9.90 ± 0.02 ^a^
Consistency	9.00 ± 0.03 ^d^	9.20 ± 0.02 ^c^	9.25 ± 0.01 ^c^	9.50 ± 0.03 ^b^	9.70 ± 0.02 ^a^
Sweet taste	8.50 ± 0.01 ^c^	9.60 ± 0.02 ^a^	9.60 ± 0.05 ^a^	8.75 ± 0.01 ^b^	8.50 ± 0.01 ^c^
Sour taste	4.80 ± 0.05 ^e^	6.30 ± 0.02 ^d^	6.60 ± 0.02 ^c^	9. 00 ± 0.03 ^b^	9.50 ± 0.05 ^a^
Flavour	6.80 ± 0.05 ^e^	8.43 ± 0.04 ^d^	8.65 ± 0.02 ^c^	9.90 ± 0.05 ^a^	9.20 ± 0.03 ^b^
Acceptability	7.80 ± 0.05 ^e^	8.25 ± 0.03 ^d^	8.75 ± 0. 00 ^c^	9.80 ± 0.02 ^a^	9.20 ± 0.02 ^b^

Note: Values are presented as the mean of three determinations ± standard deviation. Within each row, values associated with the same lowercase letters are not significantly different at *p* ≤ 0.05 according to Tukey’s test.

## Data Availability

The original contributions presented in this study are included in the article. Further inquiries can be directed to the first and corresponding author.
